# Focused screening reveals functional effects of microRNAs differentially expressed in colorectal cancer

**DOI:** 10.1186/s12885-019-6468-5

**Published:** 2019-12-21

**Authors:** Danuta Sastre, João Baiochi, Ildercilio Mota de Souza Lima, Felipe Canto de Souza, Amanda Cristina Corveloni, Carolina Hassib Thomé, Vitor Marcel Faça, Josiane Lilian dos Santos Schiavinato, Dimas Tadeu Covas, Rodrigo Alexandre Panepucci

**Affiliations:** 1 Laboratory of Human and Medical Genetics, Federal University of Pará, Rua Augusto Corrêa, 01. Guamá., Belém, Pará CEP 66075-110 Brazil; 2 Laboratory of Functional Biology (LFBio), Center for Cell-Based Therapy (CTC), Regional Blood Center, Ribeirao Preto Medical School, University of São Paulo (USP), R. Ten. Catão Roxo, 2501., Ribeirão Preto, SP 14051-140 Brazil; 3 Department of Biochemistry and Immunology, Ribeirão Preto Medical School, University of São Paulo (USP), Av. Bandeirantes, 3900 - Vila Monte Alegre, Ribeirão Preto, SP 14049-900 Brazil

**Keywords:** Colorectal Cancer, microRNAs, miR-101-3p, Proliferation, Cell death, MCL-1, miR-22-3p, miR-24-3p, Cancer stem cell, Apoptosis

## Abstract

**Background:**

Colorectal cancer (CRC) is still a leading cause of death worldwide. Recent studies have pointed to an important role of microRNAs in carcinogenesis. Several microRNAs are described as aberrantly expressed in CRC tissues and in the serum of patients. However, functional outcomes of microRNA aberrant expression still need to be explored at the cellular level. Here, we aimed to investigate the effects of microRNAs aberrantly expressed in CRC samples in the proliferation and cell death of a CRC cell line.

**Methods:**

We transfected 31 microRNA mimics into HCT116 cells. Total number of live propidium iodide negative (PI-) and dead (PI+) cells were measured 4 days post-transfection by using a high content screening (HCS) approach. HCS was further used to evaluate apoptosis (via Annexin V and PI staining), and to discern between intrinsic and extrinsic apoptotic pathways, by detecting cleaved Caspase 9 and 8, respectively. To reveal mRNA targets and potentially involved mechanisms, we performed microarray gene expression and functional pathway enrichment analysis. Quantitative PCR and western blot were used to validate potential mRNA targets.

**Results:**

Twenty microRNAs altered the proliferation of HCT116 cells in comparison to control. miR-22-3p, miR-24-3p, and miR-101-3p significantly repressed cell proliferation and induced cell death. Interestingly, all anti-proliferative microRNAs in our study had been previously described as poorly expressed in the CRC samples. Predicted miR-101-3p targets that were also downregulated by in our microarray were enriched for genes associated with Wnt and cancer pathways, including MCL-1, a member of the BCL-2 family, involved in apoptosis. Interestingly, miR-101-3p preferentially downregulated the long anti-apoptotic MCL-1 L isoform, and reduced cell survival specifically by activating the intrinsic apoptosis pathway. Moreover, miR-101-3p also downregulated IL6ST, STAT3A/B, and MYC mRNA levels, genes associated with stemness properties of CRC cells.

**Conclusions:**

microRNAs upregulated in CRC tend to induce proliferation in vitro, whereas microRNAs poorly expressed in CRC halt proliferation and induce cell death. We provide novel evidence linking preferential inhibition of the anti-apoptotic MCL-1 L isoform by miR-101-3p and consequent activation of the intrinsic apoptotic pathway as potential mechanisms for its antitumoral activity, likely due to the inhibition of the IL-6/JAK/STAT signaling pathway.

## Background

Colorectal cancer (CRC) is still the third most common cancer worldwide despite recent advancements in screening and treatment. The American Cancer Society estimates that over 100,000 new cases were diagnosed and more than 50,000 deaths are attributed to CRC in the United States alone in 2019 [1, 2]. MicroRNAs (miRNAs) are small nucleic acids involved in the post-transcriptional regulation of gene expression, and have been implicated in the pathogenesis and prognosis of CRC [3–5].

miRNAs are usually encoded in the human genome as clusters. After nuclear processing, these molecules are exported to the cytoplasm and loaded into RNA-induced silencing complexes (RISC), directing them against binding sites in the 3′-UTR region of target mRNAs, based on the degree of complementarity. While a perfect match leads to mRNA cleavage, miRNAs with partial complementarity lead to translation blockade and/or mRNA degradation through multiple mechanisms [6]. In either case, miRNAs predominantly act to decrease target mRNA levels [7]. Since a miRNA:mRNA perfect match is not required for miRNA silencing of its targets, one miRNA can affect the expression of hundreds of target transcripts. Hence, deregulation of a single miRNA can lead to global alterations in gene expression in a given cell [8].

Aberrant expression of miRNAs contributes to tumorigenesis mainly by two mechanisms: repression of tumor suppressor genes or loss of repression of oncogenes [9]. In the first case, miRNAs become overexpressed and downregulate the expression of tumor suppressor genes; in the latter case, miRNAs become downregulated themselves while their oncogene targets are overexpressed due to reduced post-transcriptional silencing. This abnormal miRNA profile can facilitate proliferation and survival of tumor cells in malignancies such as CRC [10].

miRNAs controlling pluripotency of embryonic stem cells have been associated with tumorigenesis in diverse cancers, including CRC [11–13]. In fact, cancer cells and pluripotent stem cells share the ability to proliferate rapidly and virtually indefinitely [14]. Strikingly, reprograming of somatic cells into induced pluripotent stem cells share many similarities with the process of malignant transformation [15]. Therefore, miRNAs controlling stemness and differentiation of stem cells have potential to be used as targets for the study of uncontrolled proliferation in cancer. However, this has not yet been tested in the context of CRC, and functional data on the effects of these miRNAs in the survival of CRC cells is still lacking.

We hypothesized that miRNAs involved in the control of pluripotency and differentiation of stem cells can alter the proliferation and survival of CRC cells. With that in mind, we have selected a panel of 31 miRNAs that have their expression modulated during the differentiation of embryonic stem cells [16]. We then set out to identify the effects of these miRNAs on the proliferation and cell death in a human cellular model of CRC. Importantly, most of the miRNAs in this panel have been described to be differentially expressed in CRC **(**Table 1**)**. Here, we identified three miRNAs that suppressed proliferation of CRC cells while also inducing significant cell death. Microarray analysis of miR-101-3p targets revealed modulation of relevant cancer-related pathways. We also provide further evidence that loss of miR-101-3p expression in colorectal cancer can confer proliferative advantage to malignant cells via inhibition of intrinsic apoptotic pathway.

**Table 1:** miRNAs differentially expressed in CRC and their function in embryonic stem cells (ESC)

microRNA	Expression in CRC	Tissue	Reference	Function in ESC [16]
hsa-miR-17-3p	Up	Tumor tissue	[91]	Pluripotency
	Up	Serum	[92]	
	Up	Tumor tissue	[93]	
	Up	Tumor tissue	[34]	
hsa-miR-18a-5p	Up	Plasma	[94]	Pluripotency
	Up	Fixed tumor tissue	[95]	
	Up	Tumor tissue	[93]	
	Up	Tumor tissue	[34]	
	Up	Tumor tissue	[96]	
hsa-miR-18b-5p	Up	Tumor tissue	[97]	Pluripotency
	Up	Tumor tissue	[98]	
hsa-miR-19a-3p	Up	Serum	[99]	Pluripotency
	Up	Serum	[92]	
	Up	Tumor tissue	[98]	
	Up	Tumor tissue	[96]	
hsa-miR-19b-3p	Up	Tumor tissue	[96]	Pluripotency
	Up	Serum	[92]	
hsa-miR-20a-5p	Up	Tumor tissue	[93]	Pluripotency
	Up	Serum	[92]	
	Up	Fixed tumor tissue	[95]	
	Up	Tumor tissue	[96]	
hsa-miR-20b-5p	Down	Tumor tissue	[100]	Pluripotency
	Up	Tumor tissue	[34]	
hsa-miR-21-5p	Up	Serum	[99]	Differentiation
	Up	Tumor tissue	[34]	
	Up	Fixed tumor tissue	[95]	
	Up	Tumor tissue	[96]	
	Up	Tumor tissue	[101]	
hsa-miR-22-3p	Down	Tumor tissue	[102]	Differentiation
	Down	Tumor tissue	[103]	
	Up	Tumor tissue	[101]	
hsa-miR-23a-3p	Up	Tumor tissue	[104]	Differentiation
	Up	Tumor tissue	[105]	
hsa-miR-24-3p	Up	Serum	[92]	Differentiation
	Down	Plasma	[36]	
hsa-miR-27a-3p	Up	Tumor tissue	[106]	Differentiation
	Down	Tumor tissue	[107]	
hsa-miR-29a-3p	Up	Tumor tissue	[96]	Differentiation
	Up	Fixed tumor tissue	[95]	
hsa-miR-29b	Down	Tumor tissue	[108]	Pluripotency
	Up	Tumor tissue	[96]	
hsa-miR-30a-5p	Down	Tumor tissue	[96]	Differentiation
	Down	Tumor tissue	[100]	
hsa-miR-92a-3p	Up	Tumor tissue	[93]	Pluripotency
	Up	Plasma	[93]	
	Up	Tumor tissue	[34]	
	Up	Fixed tumor tissue	[95]	
hsa-miR-101-3p	Down	Tumor tissue	[109]	Pluripotency
	Down	Serum	[110]	
	Down	Tumor tissue	[111]	
hsa-miR-106a-5p	Up	Tumor tissue	[93]	Pluripotency
	Up	Tumor tissue	[34]	
	Up	Tumor tissue	[96]	
hsa-miR-145-5p	Down	Tumor tissue	[112]	Differentiation
	Down	Tumor tissue	[34]	
	Down	Tumor tissue	[96]	
hsa-miR-181d-5p	Up	Tumor tissue	[105]	Differentiation
	Up	Fixed tumor tissue	[113]	
hsa-miR-222-3p	Up	Tumor tissue	[93]	Differentiation
	Up	Plasma	[93]	
hsa-miR-302a-3p	Down	CRC cell lines	[114]	Pluripotency
hsa-miR-302a-5p	Unknown	–	–	Pluripotency
hsa-miR-302b-3p	Unknown	–	–	Pluripotency
hsa-miR-302b-5p	Unknown	–	–	Pluripotency
hsa-miR-302c-3p	Down	Plasma	[115]	Pluripotency
hsa-miR-302d-3p	Unknown	–	–	Pluripotency
hsa-miR-363-3p	Down	Tumor tissue	[116]	Pluripotency
hsa-miR-371a-3p	Unknown	–	–	Pluripotency
hsa-miR-372-3p	Up	Fixed tumor tissue	[113]	Pluripotency
hsa-miR-373-3p	Up	Fixed tumor tissue	[113]	Pluripotency
	Up	Fixed tumor tissue	[117]	

*CRC* Colorectal cancer, *ESC* Embryonic stem cells

## Methods

### Cell culture and miRNA transfection

Human CRC cell line HCT116 (ATCC® CCL-247™) was cultivated using DMEM high-glucose supplemented with 10% FBS. Medium was changed every two days and cells were passaged by enzymatic treatment with TrypLE (ThermoFisher, Cat. No. 12604021) when 90–100% confluent. Cells were subcultured at 1:6 ratio into new flasks. HCT116 cells recapitulate many features of CRC in vitro and in vivo and are considered a suitable tool for the study of molecular characteristics of CRC in vitro [17–19].

Synthetic miRNA mimics (pre-miRs) and an unspecific control (pre-miR control) were individually transfected into HCT116 cells by reverse transfection **(**Additional file 3: Table S1**)**. Pre-miR molecules are small, double-stranded RNA molecules designed to mimic endogenous mature miRNAs. Chemical modifications induce loading of the correct strand into RISC (Additional file 3: Table S1). Upon delivery via lipofection, one strand of the pre-miR molecule is loaded into RISC complexes, where it can modulate expression of target mRNAs, mimicking the effects of native miRNAs.

In summary, 50uL of culture medium containing 8 × 10^3^ cells was added to wells of 96-well plates pre-filled with a mixture composed of 0.15 uL Lipofectamine RNAiMax (ThermoFisher, Cat. No. 13778150) and oligonucleotides in 50uL serum-free culture medium. A final concentration of 50 nM of miRNAs or siRNA against Ubiquitin C (siUBC; Dharmacon, Cat. No. M-019408-01) were used. Alternatively, HCT116 were transfected with 0.2 μL/well of Lipofectamine 2000 (ThermoFisher, Cat. No. 25887), following manufacturer’s instructions. Medium was changed 24 h post-transfection, and cells were kept in culture for 4 additional days for proliferation assay. For gene expression analysis, 8 × 10^4^ cells were seeded in 6-well plates 18-24 h before miRNA transfection. Transfection protocol was adjusted for a final volume of 1 mL. Cells were collected 72 h post-transfection for RNA extraction, used for qPCR and microarray analyses.

### Proliferation, apoptosis, and cell death assays

For proliferation assay, medium was removed after 4 days in culture and replaced by a 1.25 μg/mL solution of membrane-impermeant Propidium Iodide (PI) and 1uM of the membrane-permeant Hoechst 33342 (Hoechst) DNA stains, in final volume of 100 μL PBS. After an incubation period of 10 min, images were acquired using a High Content Screening automated fluorescence microscopy platform (ImageXpress; Molecular Devices Inc.), under 10X objective. Excitation and emission channels used were 377/447 nm and 531/593 nm for PI and Hoechst, respectively. Nuclei of live cells (i.e. with intact membranes) were stained only by Hoechst, whereas nuclei of dead cells were stained by PI as well. For each well of a 96-well plate, nine fields were acquired and all cells within this area were quantified. For confirmatory apoptosis assays, cells were incubated with 0.5 μL/well of viability stain Annexin V conjugated with Alexa Fluor 647 (ThermoFisher, Cat. No. A23204). After an incubation period of 2 h at 37 °C, a solution containing Hoechst and PI was added to the wells and incubated for 50 min. Images were acquired using a High Content Screening automated fluorescence microscopy platform (ImageXpress; Molecular Devices Inc.), under 10X objective.

### Immunocytochemistry

Cells grown in 96-well plates were fixed and permeabilized with a 2% formaldehyde solution in methanol, for 20 min at − 20 °C. Quenching of formaldehyde was achieved by incubation for 15 min with a 0.1 M glycine solution and blocked with a 1% FBS solution for 30 min. Cells were then incubated for 1 h at room temperature with primary antibodies rabbit anti-Caspase-8 IgG mAb (1:400, cleaved caspase-8, Cell Signaling Technology, Cat. No. 9496) or rabbit-anti-Caspase-9 IgG mAb (1:400, cleaved caspase-9, Cell Signaling Technology, Cat. No. 9502), followed by incubation for 45 min with a solution containing secondary antibody donkey anti-Rabbit DyLight 488 (1:300, ThermoFisher, Cat. No. 35553), as well as nuclear and cytoplasmic dyes Hoechst or CellMask Blue (2.5 μg/mL, ThermoFisher, Cat. No. H32720).

### Image acquisition and analysis

For each well of a 96-well plate used, nine fields of view were imaged at 10X objective, and a constant exposure time was used for treatment and controls for every channel, respecting the dynamic range of the camera. Raw image files were inspected and analyzed using the Multi Wavelength Cell Scoring application module of the MetaXpress software (Molecular Devices). Briefly, nuclear and cytoplasm segmentation was obtained by automated intensity thresholding of Hoechst (Apoptosis and Cell Death assay) or CellMask Blue (for Caspase 8/9 assays) signal, respectively. Percentages of positive cells for the evaluated marker were then quantified. ANOVA test with Dunnett post-test was used to detect differences between miRNAs mimics and control PMC.

### RNA extraction and RT-qPCR

Total RNA was extracted from cells 72 h post-transfection using Trizol reagent (ThermoFisher, Cat. No. 15596018), following manufacturer’s instructions. cDNA was generated by reverse transcription using 1 μg of RNA as starting material following manufacturer’s instructions for High Capacity cDNA Reverse Transcription kit (ThermoFisher, Cat. No. 4368814). Real-time qPCR reactions were performed using SYBR Green PCR master mix (ThermoFisher, Cat. No. 4309155) and primers designed in house **(**Additional file 3: Table S2**)** using 10 ng of cDNA. Relative gene expression was calculated using the 2^-∆∆CT^ method. *GAPDH* was the normalizer housekeeping gene and Control was used as reference sample. All experiments were performed in 3 biological replicates. t-test was used to detect differences between treatments and control.

### Oligonucleotide microarray and Bioinformatic analyses

Whole Human Genome Microarray Kit 4x44K (Agilent, Cat. No. G4112F) was used to detect mRNA expression levels in cells transfected with control and miR-101-3p transfected HCT116 cells, following manufacturer’s instructions. Differential expression of 41,000+ unique transcripts was analyzed using bioinformatics package Bioconductor [20]. Results were normalized using LIMMA package [21]. Transcripts were considered differentially expressed when fold change was higher than 0.5 and *p* < 0.05, using moderate T test. False Discovery Rate (FDR) test was used to adjust *p* values.

Predicted targets of miR-101-3p were obtained from the TargetScan database [22]. In order to carry a pathway enrichment analysis, we used the whole set of predicted targets that were also downregulated by miR-101-3p in our microarray analysis. This set of transcripts were analyzed using the Database for Annotation, Visualization and Integrated Discovery (DAVID) [23], restricting the analysis to pathway data from the Kyoto Encyclopedia of Genes and Genomes (KEGG) [24].

### Protein extraction and quantification

A total of 2 × 10^6^ transfected cells grown in 75 cm^2^ flasks were washed with PBS and disrupted in lyses buffer (20 mM Tris-HCl (pH 7.5), 150 mM NaCl, 1 mM Na_2_EDTA, 1 mM EGTA, 1% Triton X-100, 2.5 mM sodium pyrophosphate, 1 mM β-glycerophosphate, 1 mM Na_3_VO_4_ and1 μg/ml leupeptin). After three sonication cycles at 45 W for 5 min each (samples were kept on ice between sonication cycles) in a sonicator bath of 800 mL (Unique, São Paulo, Brazil), the samples were centrifuged at 20000 x g for 30 min at 4 °C. The protein concentration was determined by the Bradford method (Bio-Rad).

### Western blotting

Proteins were submitted to SDS–PAGE and electrotransferred to PVDF membranes (GE Lifesciences). Membranes were blocked with 5% non-fat dry milk in 0.1% Tween-TBS and incubated with the primary antibody. Rabbit anti-MCL-1 (sc-958) and mouse anti-β-actin (sc-81,178) were purchased from Santa Cruz Biotechnology. After 1 h of incubation with horseradish peroxidase-conjugated goat anti-rabbit IgG (Cell Signaling, Cat. No. 7074) or horse anti-mouse IgG (Cell Signaling, Cat. No. 7076) secondary antibodies, the antibody-protein complex was detected using ECL Western Blotting Detection Reagents (GE Lifesciences) using a CCD-Camera (Image QuantLAS 4000 mini, Uppsala, Sweden). Densitometric analysis was performed using the ImageJ software [25], and bands were normalized to constitutive proteins (*β*-actin).

## Results

### microRNAs induce or halt proliferation of colorectal cancer cells

Several miRNAs have been reported to be differentially expressed in CRC tissue when compared to normal adjacent tissues, or between the serum of CRC patients and healthy controls. However, discrepant results are often found by different authors for several microRNAs **(**Table 1**)**. Additionally, the functional outcomes of up- and downregulation of specific miRNAs in colorectal cancer cells remain to be fully evaluated.

**Fig. 1:**
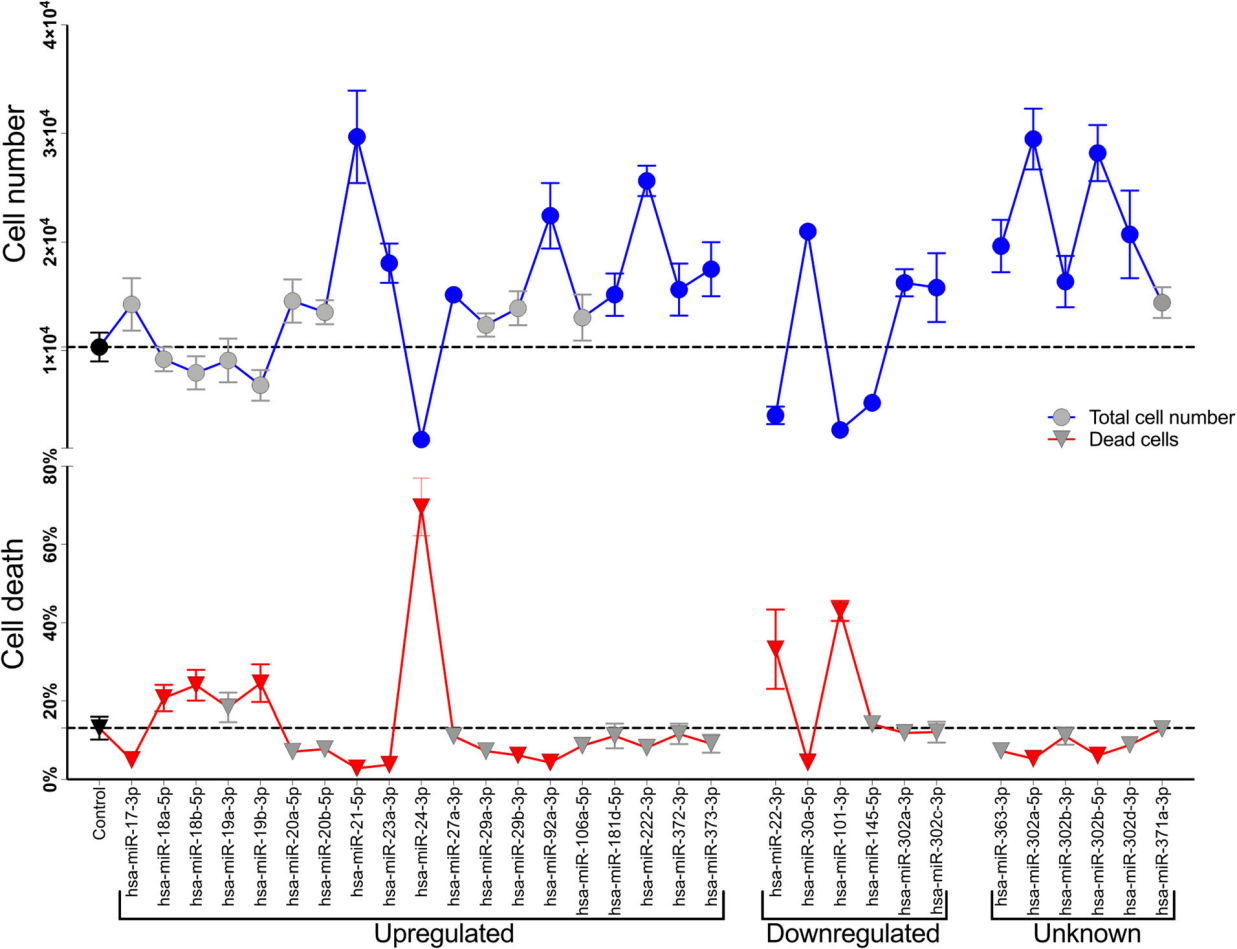
miRNAs differentially expressed in CRC modulate proliferation of HCT116 cells. HCT116 cells were transfected with 31 miRNA mimics and a control miRNA, cultured for 4 days in 96-well plates, stained with propidium iodide (PI) and Hoechst 33342, and submitted to quantitative automated fluorescence microscopy. Graph shows total cell numbers (Hoechst segmented nuclei) as blue circles and the percentage of dead cells (PI+) as red triangles. Data is expressed as mean ± SD and symbols in gray indicate no statistically significant differences (P < 0.05, unpaired one-tailed non-parametric Mann-Whitney test; *n* = 4 replica wells, 9 sites/well). The dotted line indicates the mean value obtained for the controls (black symbols). Differential expression in CRC, as found in the literature (Table 1), was used to group miRNAs and is indicated at the bottom of the figure

To investigate the effects of miRNAs on the proliferation and survival of CRC cells, we performed a focused screen in HCT116 cell line. Cells were transfected with 31 synthetic miRNA duplex mimics (pre-miR) and cultured for 4 days. Total number of live and dead cells was determined by fluorescence staining of transfected cells and imaging using a High content screening (HCS) platform. Image analysis of transfected cells allowed us to simultaneously identify miRNAs affecting cell proliferation and/or death of CRC cells **(**Fig. 1; Additional file 1).

Four days following transfection of miRNAs mimics on HCT116 cells, 16 of the miRNAs induced proliferation significantly (i.e. higher total cell counts, as compared to control), while only 4 repressed it. On the other hand, 8 miRNAs reduced cell death (i.e. lower percentage of dead cells, as compared to control), while 6 induced it.

Eight miRNAs described as upregulated in CRC tissues or serum samples induced significant increase in cell proliferation (miR-21-5p, −23a-3p, −27a-3p, −92a-3p, −181d-5p, − 222-3p, − 372-3p, and − 373-3p), whereas only one miRNA upregulated in serum, but downregulated in plasma, inhibited proliferation (miR-24-3p). Cancer and pluripotency related miRNAs belonging to clusters miR-106a~ 363, miR-17~92 and miR-302 induced significant increase in proliferation. Notably, mimics of miR-22-3p, miR-24-3p, and miR-101-3p, all described as CRC-downregulated miRNAs, simultaneously reduced cell proliferation and induced cell death, significantly, when compared to control. We decided to focus on miR-101-3p for further experiments due to described involvement of this miRNA in CRC.

### miR-101 induces apoptosis in HCT116 cells.

**Fig. 2:**
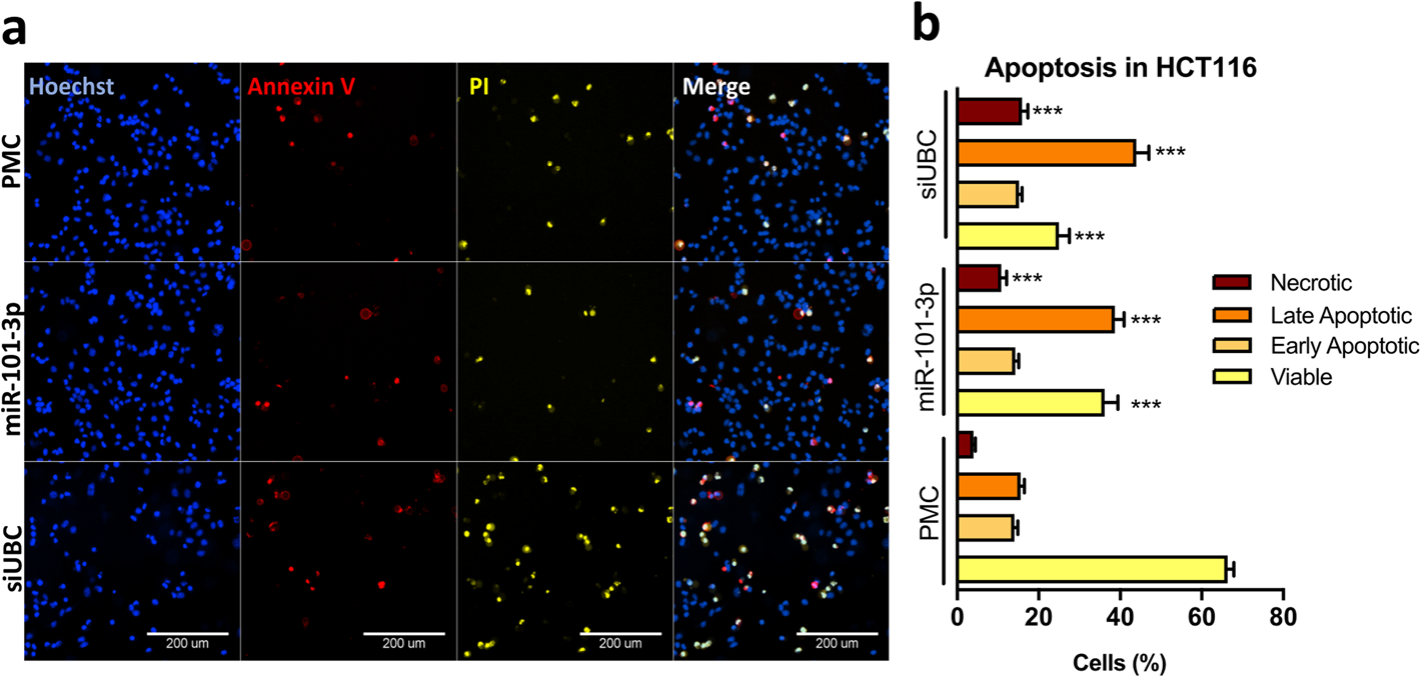
miR-101-3p induces apoptosis in HCT116 cells. Cells were transfected with control miRNA (PMC), miR-101-3p or a control lethal siRNA against ubiquitins (siUBC), cultured for 72 h in 96-well plates, stained for apoptotic markers, and submitted to quantitative automated fluorescence microscopy. (**a**) Representative images of HCT116 cells stained with Annexin V (red), Propidium Iodide (PI, yellow) and Hoechst (blue). (**b**) Quantification of apoptotic markers separated cells into four populations: viable (Hoechst only), early apoptotic cells (double positive for Annexin V and Hoechst only), late apoptotic (triple positive for PI, Annexin V, and Hoechst), and necrotic (double positive for PI and Hoechst only). Quantification plots show significant increase in necrotic and late apoptotic cells with decrease in viable cells after treatment with miR-101-3p in comparison to control PMC (*P* < 0.05, unpaired one-tailed non-parametric Mann-Whitney test; *n* = 5 replica wells, 9 sites/well)

**Fig. 3:**
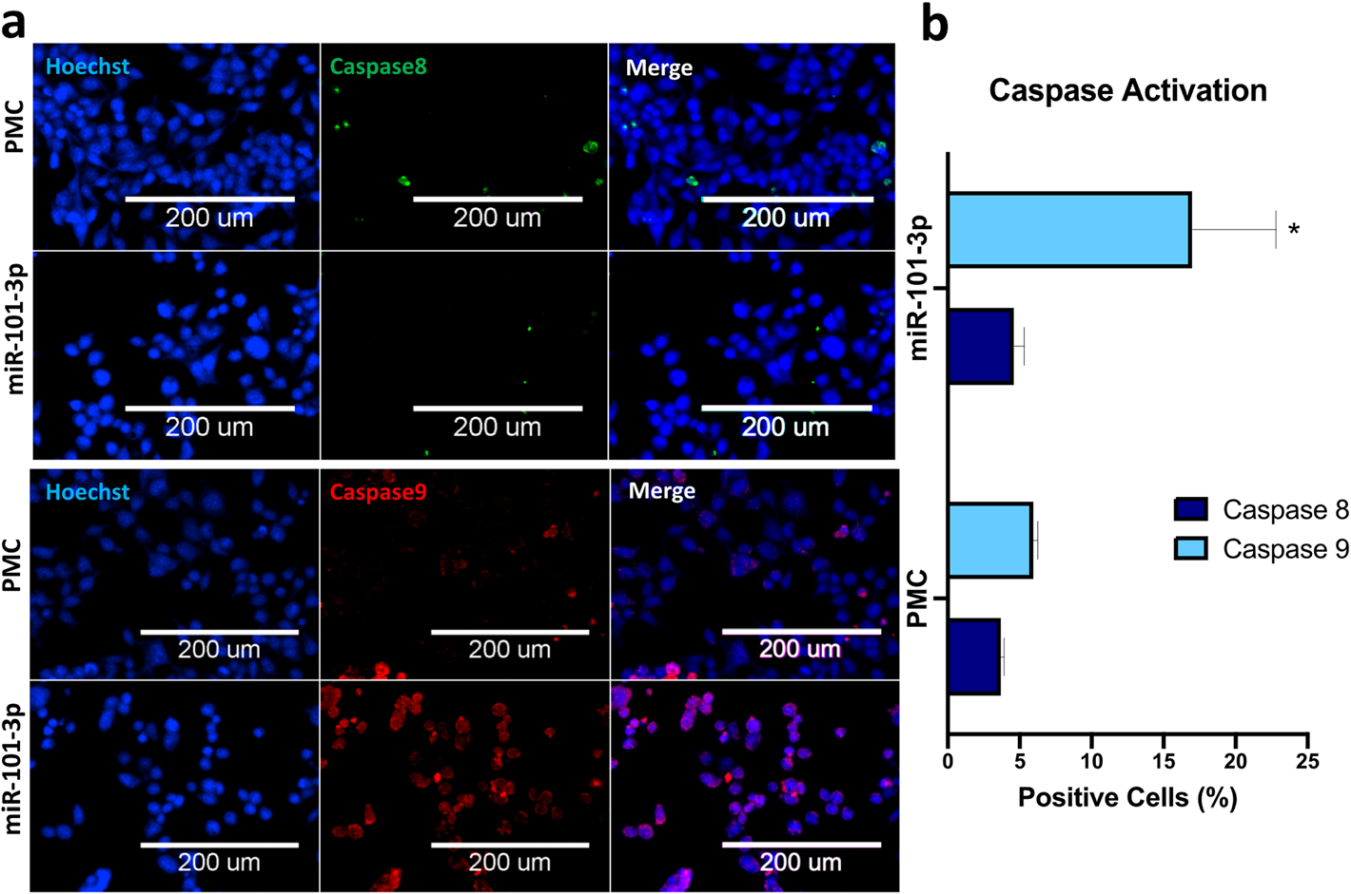
miR-101 activates intrinsic apoptosis pathway in HCT116 cells. (**a**) Representative images of HCT116 cells transfected with miR-101-3p, control PMC or lethal siRNA siUBC and stained with Hoechst (blue) and for cleaved caspase 8 (green) or cleaved caspase 9 (red). (**b**) Quantification plots showing a statistically significant increase in the percentages of Caspase 9 positive cells following transfection with miR-101-3p as compared to PMC (*P* < 0.05, unpaired one-tailed non-parametric Mann-Whitney test; ***n*** = 3 replica wells, 9 sites/well)

Initial screening showed significant reduction in the number of cells treated with miR-101-3p, and an increase in propidium iodide positive (PI+) dead cells. To identify if cell death was induced via apoptosis or necrosis, we performed additional quantitative immunofluorescence experiments for fluorescently labeled Annexin V and PI **(**Fig. 2**)**. In response to miR-101-3p, we observed a marked reduction in the percentage of viable cells and increased percentages of necrotic (Annexin V- PI+) and late apoptotic (Annexin V+ PI+) cells. Additionally, miR-101-3p markedly increased the percentage of cells positive for cleaved activated caspase-9, but not for cleaved caspase-8 **(**Fig. 3**)**, indicating activation of the intrinsic apoptotic pathway.

### miR-101 downregulates signaling pathways controlling cell survival

**Fig. 4:**
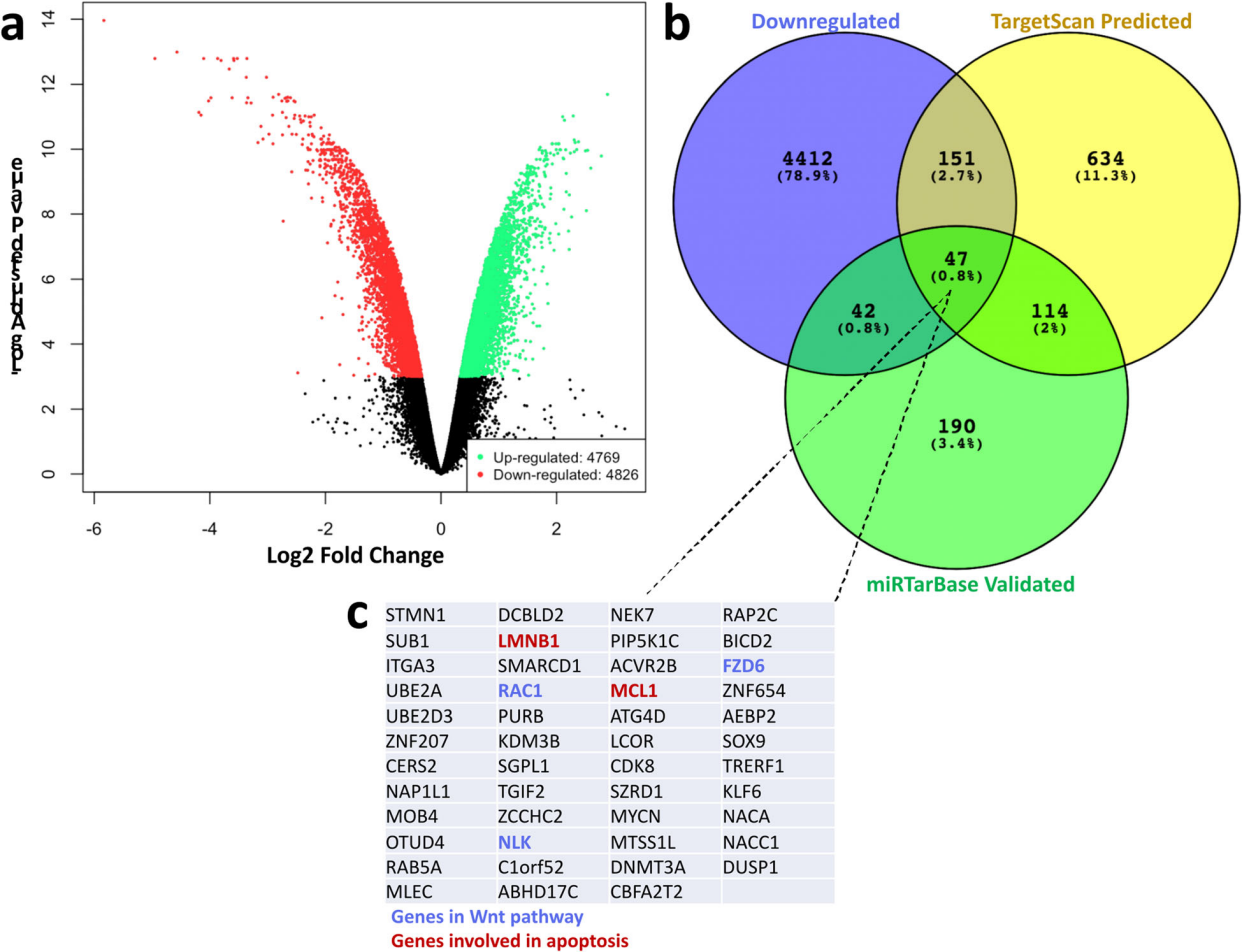
Differential gene expression in HCT116 treated with miR-101-3p. HCT116 cells were transfected with control miRNA (PMC, *n* = 3) or miR-101-3p (n = 3) and submitted to microarray for differential gene expression. (a) Volcano plot showing downregulated (red) and upregulated (green) transcripts as a function of fold-change and P-value after 3 days of treatment. (b) Venn diagram showing number and relative percentages of shared and exclusive transcripts among those experimentally identified by microarray analysis (downregulated), predicted by TargetScan or functionally validated targets identified in the miRTarBase. (c) All 47 high confidence targets predicted by TargetScan and identified as validated targets by miRTarBase that were downregulated by miR-101-3p in our microarray analysis

To identify potential post-transcriptional regulatory mechanisms mediating the observed functional effects of miR-101-3p, we performed a gene expression analysis using oligonucleotide microarray of HCT116 cells transfected with miR-101-3p mimics or a control PMC. A total of 4826 transcripts were significantly downregulated by miR-101-3p **(**Fig. 4a**)**. In silico predictions of miR-101-3p targets from the TargetScan database [22] were crossed with our experimental data to identify transcripts most likely to be directly regulated by miR-101-3p **(**Fig. 4b**)**. Twenty percent (198 out of 947) of miR-101-3p predicted targets were downregulated in HCT116 treated with miR-101-3p. Moreover, 47 of these targets had also been experimentally validated by diverse studies cataloged by miRTarBase [26] **(**Fig. 4c**)**, featuring Wnt and apoptosis-related genes. This subset of predicted target transcripts downregulated upon introduction of miR-101-3p in HCT116 CRC cells, and previously experimentally validated in independent studies, represent high-confidence targets **(**Additional file 2**)**.

In order to extract information regarding cellular processes that could be post-transcriptionally modulated by miR-101-3p, we performed a pathway enrichment analysis using Database for Annotation, Visualization and Integrated Discovery (DAVID) [23] by entering the set of predicted miR-101-3p targets that were downregulated in our microarray (198 transcripts)**.** Perhaps not surprisingly, “Pathways in Cancer” and “Wnt” had the highest enrichment of targets associated with miR-101-3p expression. Kyoto Encyclopedia of Genes and Genomes (KEGG) [24] pathway data was used to organize genes lists according to function **(**Table 2**)**.

**Table 2:** KEGG signaling pathways modulated by predicted miR-101-3p targets downregulated experimentally in HCT116 cells

Pathway and Genes	Target Count	%	*P*-Value	Benjamini
Wnt signaling pathway *CXXC4, CAMK2G, FZD4, FZD6, NLK, PLCB1, RAC1*	7	3,6	2,1E-3	2,7E-1
Melanogenesis *GNAI3, ADCY6, CAMK2G, FZD4, FZD6, PLCB1*	6	3,1	2,7E-3	1,8E-1
Pathways in cancer *CEBPA, GNAI3, ADCY6, FZD4, FZD6, ITGA3, PAX8, PLCB1, RAC1, RXRB, TCEB1*	11	5,7	4,3E-3	1,9E-1
Sphingolipid signaling pathway *GNAI3, CERS2, CERS6, PLCB1, RAC1, SGPL1*	6	3,1	6,0E-3	2,0E-1
Ubiquitin mediated proteolysis *MGRN1, TCEB1, UBE2A, UBE2D1, UBE2D3, UBE2Q1*	6	3,1	1,0E-2	2,6E-1
Phosphatidylinositol signaling system *CDS2, MTMR2, PIP5K1C, PLCB1, TMEM55A*	5	2,6	1,5E-2	3,1E-1
Transcriptional misregulation in cancer *CEBPA, DOT1L, PAX8, RXRB, SLC45A3, MYCN*	6	3,1	2,3E-2	3,9E-1
Gastric acid secretion *GNAI3, ADCY6, CAMK2G, PLCB1*	4	2,1	3,3E-2	4,7E-1
cAMP signaling pathway *GNAI3, SOX9, ADCY6, CAMK2G, PDE4A, RAC1*	6	3,1	4,2E-2	5,1E-1
Proteoglycans in cancer *GAB1, CAMK2G, CAV3, FZD4, FZD6, RAC1*	6	3,1	4,4E-2	4,9E-1
Insulin secretion *ADCY6, CAMK2G, PLCB1, KCNN3*	4	2,1	4,9E-2	4,9E-1
Gap junction *GNAI3, ADCY6, GJA1, PLCB1*	4	2,1	5,3E-2	4,9E-1
Circadian entrainment *GNAI3, ADCY6, CAMK2G, PLCB1*	4	2,1	6,4E-2	5,3E-1
Inflammatory mediator regulation of TRP channels *ASIC1, ADCY6, CAMK2G, PLCB1*	4	2,1	6,9E-2	5,3E-1
Sphingolipid metabolism *CERS2, CERS6, SGPL1*	3	1,6	7,6E-2	5,4E-1
Cholinergic synapse *GNAI3, ADCY6, CAMK2G, PLCB1*	4	2,1	9,2E-2	5,9E-1

**Fig. 5:**
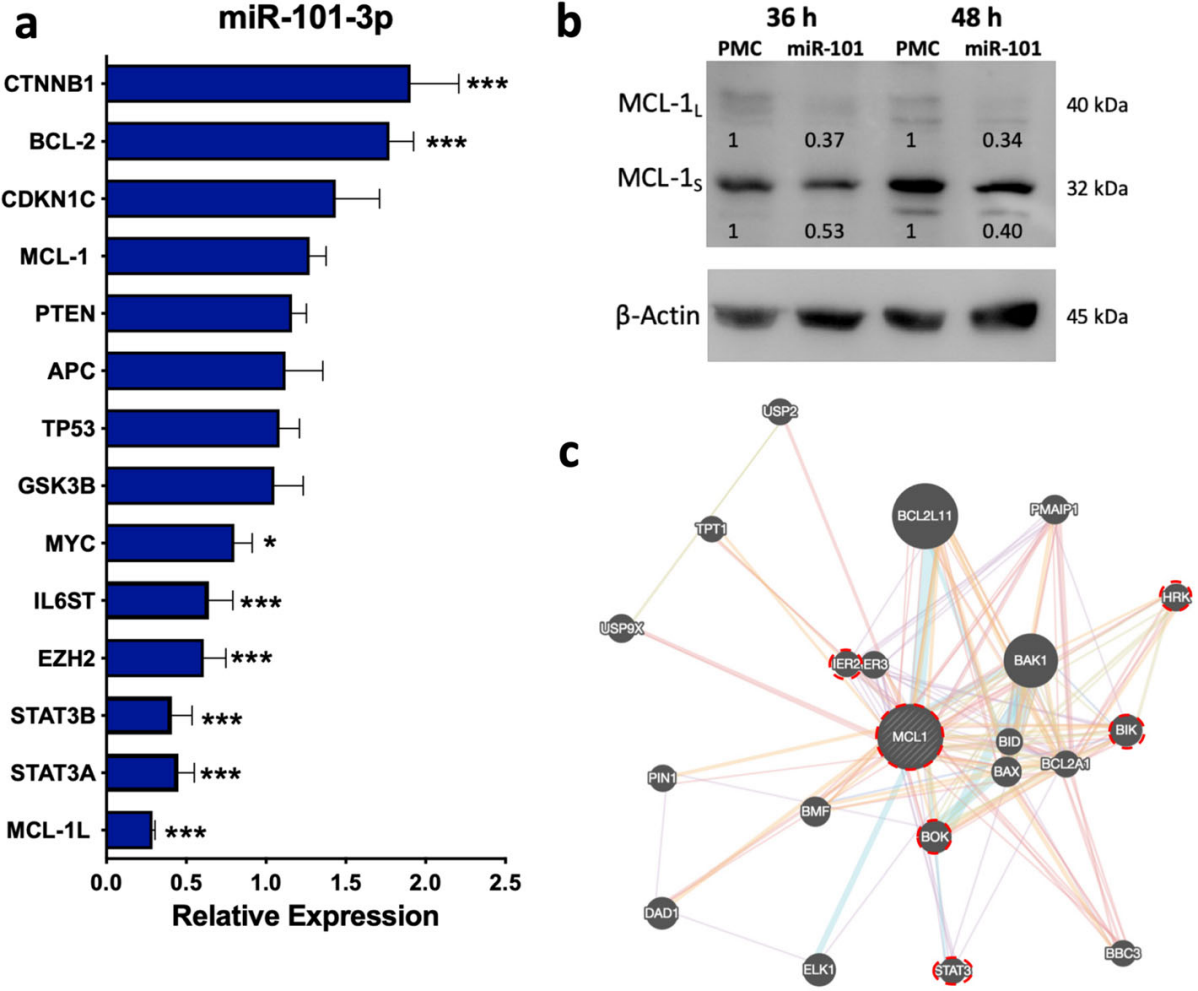
Genes downregulated by miR-101-3p in HCT116. HCT116 cells were transfected with control miRNA (PMC) and miR-101-3p and submitted to qPCR (n = 3) or Western Blot (for 36 or 48 h) analysis. (a) Expression of potential direct and indirect miR-101-3p targets in miR-101-3p transfected HCT116 cells, relative to control (set as 1). Data is expressed as mean ± SD (n = 3). * *p* < 0.01, **p < 0.001, *** *p* < 0.0001; unpaired two-tailed t-test. (b) Western blot image showing band densitometry quantification of the long anti-apoptotic (MCL-1_L_) and short pro-apoptotic (MCL-1_S_) protein isoforms in HCT116 cells following transfection with miR-101-3p, relative to PMC-transfected cells (normalized for protein input, based on ß-actin bands). (c) MCL-1 protein interaction network. Red circles indicate genes downregulated by miR-101-3p in our microarray analysis

Next, we analyzed the expression of several putative miR-101-3p direct and indirect targets in HCT116 cells (Fig. 5a). First, we analyzed the expression of several oncogenes and cell cycle related transcripts. Levels of tumor suppressors Phosphatase and Tensin Homolog (*PTEN*) and Cyclin Dependent Kinase Inhibitor 1C (*CDKN1C*) did not differ significantly from control treated cells. However, miR-101-3p expression inhibited v-myc myelocytomatosis viral oncogene homolog (*MYC*) mRNA, which can help explain, at least partially, the observed halt in cell proliferation of transfected HCT116 cells. Similarly, we observed repression of Enhancer of Zeste 2 Polycomb Repressive Complex 2 Subunit (*EZH2*), which is a predicted target of miR-101-3p that has been linked to oncogenesis in CRC [27, 28]. Analysis of canonical Wnt pathway genes reveled upregulation of ß-catenin mRNA (*CTNNB1*) whereas Glycogen Synthase Kinase 3 Beta (*GSK3B*) and Adenomatous Polyposis Coli (*APC*) remained unaltered, in spite of both *GSK3B* and *APC* being predicted targets of miR-101-3p.

We found that Myeloid cell leukemia-1 (*MCL1*), a member of the BCL-2 family of tumor suppressors and a predicted target of miR-101-3p, was downregulated in our microarray data. *MCL1* gene encodes three isoforms: one long, anti-apoptotic MCL-1L, and two shorter pro-apoptotic MCL-1S and MCL-1ES [29]. Downregulation of *MCL1* mRNA observed in the microarray was confirmed by qPCR and western blot. Interestingly, qPCR revealed that the anti-apoptotic mRNA isoform of MCL-1 was specifically downregulated by miR-101-3p, while primers detecting all 3 MCL-1 variants showed no significant alteration **(MCL-1L and MCL-1, respectively,** Fig. 5a**)**. On the other hand, western blot analysis showed a reduction of MCL-1L and MCL-1S protein levels at both time points post-transfection with miR-101-3p (36 h and 48 h), however, compared to MCL-1S, MCL-1L showed a larger fold reduction at both time points **(**Fig. 5b**)**. Additional apoptosis-related genes that closely interact with MCL-1 were also downregulated by miR-101-3p in our microarray data **(**Fig. 5c**)**. To evaluate cancer stem cell related phenotypes, we used qPCR to determine the expression of STAT3A, STAT3B and IL6ST. HCT116 cells treated with miR-101-3p showed reduction of all three markers, indicating repression of cancer-initiating characteristics **(**Fig. 5a**)**.

## Discussion

In the present study, we evaluated the effects of 31 miRNAs on the proliferation and survival of a colorectal cancer cell line. Twenty miRNA mimics significantly altered HCT116 total cell numbers compared to control. Mimics of miR-22-3p, miR-24-3p, and miR-101-3p significantly reduced cell proliferation whilst inducing significant cell death when compared to control.

Differential expression of miRNAs is a common feature of many malignancies. Upregulation of oncomiRs and, conversely, downregulation of tumor suppressor miRNAs is believed to play a role in the proliferation and survival of cancer cells [9, 30]. Perhaps not surprisingly, around 30–50% of miRNAs are located at instable, cancer-associated genomic regions, and fragile sites [31, 32], which contributes to their aberrant expression profiles. miRNAs controlling pluripotency and differentiation of stem cells have been shown to be involved in tumorigenic processes and cancer stem cell derivation [33].

The panel of miRNAs tested in the present study represent miRNAs deregulated in CRC and modulated during the differentiation of embryonic stem cells [16]. It is hypothesized that during the differentiation process downregulated miRNAs are involved in the maintenance of stemness properties, whereas miRNAs upregulated are involved in the induction of differentiation. More importantly, the miRNAs tested also represent the most frequently reported as differentially expressed miRNAs in CRC **(**Table 1**)**.

Zhu and colleagues identified 38 miRNAs differentially expressed in tumor tissues of CRC patients [34]. Among the 30 miRNAs found upregulated in that study, miR-21-5p, miR-20b-5p, miR-106a-5p, miR-92a-3p and miR-17-3p were also included in our study and, except for miR-18b-5p, they all stimulated cell proliferation of HCT116 cells in our screening, albeit only significantly for miR-21-5p and miR-92-3p. On the other hand, miRNAs that significantly reduced cell proliferation and induced cell death in our study had previously been described as poorly expressed in CRC samples: miR-22-3p [35], miR-24-3p [36], and miR-101-3p [37]. Ng et al. also detected differential expression of several miRNAs tested in our screening [38]. Among the concordantly upregulated miRNAs in serum and CRC biopsies in that study, miR-92-3p and miR-222-3p were also tested in our screening and induced significant increase in proliferation of HCT116 cells.

miRNAs can be expressed from polycistronic clusters wherein several miRNAs stem from the same primary transcript [39]. In this study, we investigated miRNAs belonging to cluster miR-17~92 (miR-17-3p, −18a-5p, −19a-3p, −19b-3p, −20a-5p, −92a-3p), cluster miR-106a~ 363 (miR-18b-5p, −20b-5p, −106a-5p, − 363-3p), and cluster miR-302 (miR-302a-3p, −302a-5p, −302b-3p, −302b-5p, −302c-3p, −302d-3p). These clusters are abundantly expressed in pluripotent stem cells and are involved in stemness maintenance [40] while also being associated with deregulated proliferation and malignancies [41, 42]. Overall, the cluster miR-17~92 induced proliferation in our screening. This cluster is located at chromosome 13q31, one of the regions associated with CRC progression. Previous work has demonstrated that gain of the region containing this cluster leads to increased expression of the corresponding miRNAs in CRC tumor samples [42]. Taken together, the proliferation profile observed in our study points to a proliferative advantage for augmented expression of cluster miR-17~92 in CRC.

The pluripotency-associated cluster miR-302 induced marked proliferation of HCT116 cells in our study. Overexpression of this cluster is sufficient to reprogram somatic cells to pluripotency [43]. However, it has been suggested that although these miRNAs activate a pluripotency program in the target cells, they do so while also protecting cells from malignant transformation [44]. Previous work corroborating this idea has suggested that overexpression of miR-302 cluster actually can rescue malignant cells by reducing their proliferative profile and invasiveness [45]. A contrasting study indicates that overexpression of miR-302 cluster in cancer cells actually leads to a more invasive and undifferentiated cancer state [33]. Although our data supports the latter hypothesis, more studies in CRC models will be needed to address the context-dependent functions of miR-302 cluster in this malignancy.

miR-101-3p is one of the miRNAs downregulated during the differentiation of embryonic stem cells [16]. It markedly reduced cell proliferation and promoted cell death in our screening. Similar to our results, Chen and colleagues also demonstrated that overexpression of miR-101-3p in CRC in vitro models (HT-29 and RKO colon cancer cell lines) reduced proliferation and viability and simultaneously sensitized cells to 5-FU inhibition [46]. In fact, re-expression of miR-101-3p has been associated to in vitro sensitization of CRC cells to chemotherapy, leading to enhanced activity of paclitaxel and doxorubicin in HT-29 cells [47]. Increase in Annexin V staining and expression of caspase 9 by miR-101-3p point to activation of intrinsic apoptotic pathway as one of the tumor suppressor mechanisms in HCT116, corroborating previous studies.

miR-101-3p has been found to act as a tumor suppressor in several malignancies, such as liver [48], glioblastoma [49], breast [50], endometrial [51], and colorectal [52]. Downregulation of this miRNA is so frequently found in solid tumors that some authors propose to use miR-101-3p expression as prognostic biomarker and therapeutic target [53–56]. miR-101-3p expression is commonly found downregulated in comparison to healthy adjacent tissues and, in some instances, its expression can predict poor prognosis and overall survival in CRC [37, 46, 57].

Epigenetic factors play an important role in CRC pathogenesis and progression [58]. Here we have shown that miR-101-3p significantly repressed expression of EZH2, a member of the Polycomb Repressor Complex 2 (PCR2) which catalyzes methylation of lysine 27 of histone H3 (H3K27me3). This complex modifies the chromatin structure to favor a proliferative program by bypassing the Ink4a/Arf-pRb-p53 pathway [59]. EZH2 promotes proliferation of CRC cells, and its silencing by siRNA leads to reduced cancer cell survival [27]. Recent data has suggested the existence of a negative feedback loop between EZH2 and miR-101-3p. Treatment of CRC cells with an anti-cancer substance named methyl jasmonate led to apoptosis and inhibited expression of EZH2 while upregulated miR-101-3p expression [60]. Furthermore, EZH2 has been linked to epigenetic inactivation of WNT5A, a proposed tumor suppressor, during TGF-ß-induced epithelial-mesenchymal transition in an in vitro model of CRC [61]. EZH2 might also be involved in CRC chemotherapeutic efficacy. EZH2 repression increased the efficiency of EGFR inhibitors in vitro [28]. Similarly, Yamamoto and colleagues have shown that EZH2 expression was associated with survival in CRC patients undergoing anti-EGFR therapy [62].

Hypermethylation has been associated with CRC pathogenesis in several studies [63–65] [reviewed in [66]]. Aberrant hypermethylation phenotype of tumor suppressor genes by DNMT3a activity has been reversed by expression of miR-101-3p in a model of lung cancer, where DNMT3 repression led to promoter hypomethylation and re-expression of tumor suppressor CDH1 [67]. Perhaps not surprisingly, our microarray data revealed downregulation of both DNMT3a and DNMT3b in HCT116 cells treated with miR-101-3p mimics. Similarly, Toyota and colleagues demonstrated that miR-34b and miR-34c were epigenetically silenced in HCT116 cells, and its expression could be rescued by treatment with 5-aza-2′-deoxycytidine, a DNA demethylation agent. Moreover, they showed that CpG methylation of miR-34b/c was a common feature of different CRC lines [68]. Although authors did not investigate the methylation levels of miR-101-3p locus, CpG methylation represents a possible mechanism for repression of other miRNAs in CRC.

Our microarray data helps shed light on the involvement of Wnt pathway in CRC. Inhibition of Wnt pathway results in reduced proliferation in several cancers, including CRC [69]. We found ß-catenin mRNA, the main mediator of canonical Wnt pathway, to be overexpressed in HCT116 cells in response to miR-101-3p mimics. However, overexpression of *CTNNB1* as measured by qPCR could reflect the mutated status of this gene in HCT116 cell line. Mouradov and colleagues performed an extensive whole-exome sequencing and SNP microarray analysis of 70 CRC cell lines, which revealed *CTNNB1* mutated status of several of them, including HCT116 [70]. It is interesting to speculate that miR-101-3p may interfere with the non-canonical Wnt pathway, given that genes downregulated in our microarray most likely reflect this hypothesis (*CXXC4, CAMK2G, FZD4, FZD6, NLK, PLCB1, RAC1*). For instance, expression of Nemo-like kinase (NLK) has been demonstrated to be necessary for cell cycle progression in CRC in vitro [71].

In addition to inhibiting cell proliferation, miR-101-3p also remarkably induced cell death in treated cells. Several pathways have been implicated in the induction of apoptosis by miR-101-3p in different cancer cell models [72–74]. Microarray analysis provided some clues on what genes can be modulated in order to warrant such effect on cell survival. MCL-1 is a member of the BCL-2 superfamily of apoptosis regulators, and it is one of the most frequently amplified genes in cancers [75]. MCL-1 gene encodes three isoforms: the long, anti-apoptotic MCL-1L, and two shorter pro-apoptotic MCL-1S and MCL-1ES [29]. MCL-1 amplification accounts for resistance to the BCL-2/BCL-xL inhibitor ABT-737 [76–78]. MCL-1 associates with mitochondrial membrane-associated proteins, Bak and Bax, preventing them from heterodimerizing with apoptotic members of the BCL-2 family to promote apoptosis cascade [79]. More strikingly, a recent study has demonstrated that degradation of MCL-1 is necessary for effective therapeutics against CRC [80]. Similarly, inhibition of MCL-1 by miR-101-3p has been implicated in the apoptosis-inducing effect of anti-cancer drug doxorubicin in hepatocellular carcinoma [81]. A similar inhibitory mechanism between miR-101-3p and MCL-1 has been reported for endometrial cancer as well [51]. Given that the shift to a high MCL-1S/L ratio is associated with increased sensitivity of cancer cells to apoptotic stimuli, specifically through the mitochondrial intrinsic apoptotic pathway [82], the preferential inhibition of the anti-apoptotic MCL-1L isoform, and the concomitant and specific increase in caspase 9 positive cells observed in our study highlights a novel mechanism by which miR-101-3p can induce apoptosis, and points to a possible therapeutic target for oligonucleotide-based therapies.

HCT116 cells are one of the CRC models widely used and validated for its ability to recapitulate CRC features in vitro [17]. Berg et al. profiled the RNA and protein expression of 34 CRC cell lines, including HCT116, classifying them according to the degree of differentiation between colon-like and undifferentiated lines [19]. According to this classification, HCT116 is an undifferentiated cell line, meaning that they differentially express stemness-related genes, including miRNAs. From the 31 miRNAs in our study, 25 were described as differentially expressed in colon-like and undifferentiated CRC lines by Berg at al. Perhaps not surprisingly, miR-101-3p is downregulated in undifferentiated CRC lines which include HCT116. Moreover, all upregulated miRNAs in undifferentiated lines evaluated by Berg et al. are associated with pluripotency ([16], Table 1).

In addition to its involvement in proliferation and apoptosis, miR-101-3p repressed characteristics of cancer stem cells (CSC) in HCT116. For instance, cells transfected with miR-101-3p showed marked reduction in mRNA for STAT3A, STAT3B and IL6ST. CSC are characterized by higher expression of IL-6, IL-6R, IL6ST, and phosphorylated STAT3 [83]. STAT3 can be activated by IL-6 and its persistent activation contributes to CRC tumor growth and proliferation [84]. STAT3 targets several genes involved in oncogenesis, including members of the BCL2 family (MCL-1 included), IL-6, IL-6R, PI3K, AKT, pluripotency-related factors (such as Sox2, Nanog, and MYC), and genes involved in epithelial-mesenchymal-transition (EMT), such as Twist, Zeb1, vimentin and many metalloproteinases [85, 86]. Moreover, IL-6 can induce anti-apoptotic MCL-1L accumulation, a process dependent on the JAK/PI3K/AKT/CREB signal transduction pathway [87]. Finally, miR-101-3p can inhibit IL-6/JAK2/STAT3 signaling by targeting JAK2 [88] or ROCK1 [89]. Taken together, the results indicate that miR-101-3p inhibits CSC phenotypes and reduces MCL-1L levels by interference with IL-6/JAK2/STAT3 signaling in HCT116 cells.

Overall, the cell proliferation profile observed in our model of CRC points to an interesting tendency: miRNAs overexpressed in CRC augment cell proliferation and, conversely, miRNAs poorly expressed in CRC reduce cell proliferation and survival. Additionally, microRNAs characteristic of pluripotent stem cells tend to confer a proliferative advantage to CRC cells. This phenomenon suggests the existence of potential functional advantages of the differential expression of miRNAs observed in colorectal cancer. Since selective pressure within tumor tissue favors accumulation of genetic alterations that support survival [90], it is tempting to speculate that miRNAs consistently described as downregulated in CRC could have been selectively repressed due to their effects on proliferation, such as seen in our study.

## Conclusions

Taken together, the results provide additional evidence of functional outcomes resulting from differential expression of miRNAs in CRC. Additional studies will be necessary to elucidate the mechanisms by which miRNAs differentially expressed in CRC promote these effects on proliferation, and the present study points to interesting miRNAs to pursuit. Additionally, miR-101-3p appears to target multiple transcripts that act synergistically to promote cell death and halt proliferation of CRC cells in vitro, by inhibiting Wnt and IL-6/JAK2/STAT3 signaling pathways. More specifically, we provide novel evidence linking preferential inhibition of the anti-apoptotic MCL-1 L isoform by miR-101-3p, and consequent activation of the intrinsic apoptotic pathway, as a potential mechanisms for the antitumoral activity of this miRNA in CRC.

## Supplementary information

**Additional file 1.** miRNA screening data and statistical analyses

**Additional file 2.** Microarray data and statistical analyses

**Additional file 3 Table S1**. Synthetic miRNA mimics access number and sequence. **Table S2**. Primers used for RT-PCR.

## Abbreviations

5-FU: 5-fluouracil
AKT: Serine/threonine kinase
BCL-2: B-cell lymphoma-2
CRC: Colorectal cancer
CSC: Cancer stem cell
DAVID: Database for annotation, visualization and integrated discovery
EGFR: Epidermal growth factor receptor
EMT: Epithelial-mesenchymal-transition
EZH2: Enhancer of zeste 2 polycomb repressive complex 2 subunit
IL6ST: Interleukin 6 Signal Transducer
JAK2: Janus kinase 2
KEGG: Kyoto Encyclopedia of genes and genomes
MCL-1: Myeloid cell leukemia-1
miR: microRNA
miRNA: microRNA
NLK: Nemo-like kinase
RISC: RNA-induced silencing complex
ROCK1: Rho-associated protein kinase 1
STAT3: Signal transducer and activator of transcription 3
