# Supplementary material for: Focused screening reveals functional effects of microRNAs differentially expressed in colorectal cancer
Source: BMC Cancer. 2019 Dec 21;19:1239. doi: 10.1186/s12885-019-6468-5 (PMC6925883; doi:10.1186/s12885-019-6468-5)
Supplement: Supplementary file 3 — Additional file 3 Table S1. Synthetic miRNA mimics access number and sequence. Table S2. Primers used for RT-PCR. [file 12885_2019_6468_MOESM3_ESM.docx]

**Supplementary Table 1:** Synthetic miRNA mimics access number and sequence.

| **miRNA official name** | **miRbase Access number** | **Mature 5’- 3’ Sequence** |
| --- | --- | --- |
| hsa-miR-17-3p | MIMAT0000071 | ACUGCAGUGAAGGCACUUGUAG |
| hsa-miR-18a-5p | MIMAT0000072 | UAAGGUGCAUCUAGUGCAGAUAG |
| hsa-miR-18b-5p | MIMAT0001412 | UAAGGUGCAUCUAGUGCAGUUAG |
| hsa-miR-19a-3p | MIMAT0000073 | UGUGCAAAUCUAUGCAAAACUGA |
| hsa-miR-19b-3p | MIMAT0000074 | UGUGCAAAUCCAUGCAAAACUGA |
| hsa-miR-20a-5p | MIMAT0000075 | UAAAGUGCUUAUAGUGCAGGUAG |
| hsa-miR-20b-5p | MIMAT0001413 | CAAAGUGCUCAUAGUGCAGGUAG |
| hsa-miR-21-5p | MIMAT0000076 | UAGCUUAUCAGACUGAUGUUGA |
| hsa-miR-22-3p | MIMAT0000077 | AAGCUGCCAGUUGAAGAACUGU |
| hsa-miR-23a-3p | MIMAT0000078 | AUCACAUUGCCAGGGAUUUCC |
| hsa-miR-24-3p | MIMAT0000080 | UGGCUCAGUUCAGCAGGAACAG |
| hsa-miR-27a-3p | MIMAT0000084 | UUCACAGUGGCUAAGUUCCGC |
| hsa-miR-29a-3p | MIMAT0000086 | UAGCACCAUCUGAAAUCGGUUA |
| hsa-miR-29b-3p | MIMAT0000100 | GCUGGUUUCAUAUGGUGGUUUAGA |
| hsa-miR-30a-5p | MIMAT0000087 | UGUAAACAUCCUCGACUGGAAG |
| hsa-miR-92a-3p | MIMAT0000092 | UAUUGCACUUGUCCCGGCCUGU |
| hsa-miR-101-3p | MIMAT0000099 | UACAGUACUGUGAUAACUGAA |
| hsa-miR-106a-5p | MIMAT0000103 | AAAAGUGCUUACAGUGCAGGUAG |
| hsa-miR-145-5p | MIMAT0000437 | GUCCAGUUUUCCCAGGAAUCCCU |
| hsa-miR-181d-5p | MIMAT0002821 | AACAUUCAUUGUUGUCGGUGGGU |
| hsa-miR-222-3p | MIMAT0000279 | AGCUACAUCUGGCUACUGGGU |
| hsa-miR-302a-3p | MIMAT0000684 | UAAGUGCUUCCAUGUUUUGGUGA |
| hsa-miR-302a-5p | MIMAT0000683 | ACUUAAACGUGGAUGUACUUGCU |
| hsa-miR-302b-3p | MIMAT0000715 | UAAGUGCUUCCAUGUUUUAGUAG |
| hsa-miR-302b-5p | MIMAT0000714 | ACUUUAACAUGGAAGUGCUUUC |
| hsa-miR-302c-3p | MIMAT0000717 | UAAGUGCUUCCAUGUUUCAGUGG |
| hsa-miR-302d-3p | MIMAT0000718 | UAAGUGCUUCCAUGUUUGAGUGU |
| hsa-miR-363-3p | MIMAT0000707 | AAUUGCACGGUAUCCAUCUGUA |
| hsa-miR-371a-3p | MIMAT0000723 | AAGUGCCGCCAUCUUUUGAGUGU |
| hsa-miR-372-3p | MIMAT0000724 | AAAGUGCUGCGACAUUUGAGCGU |
| hsa-miR-373-3p | MIMAT0000726 | GAAGUGCUUCGAUUUUGGGGUGU |

**Supplementary Table 2:** Primers used for RT-PCR

| **Target gene** | **Sequence 5’- 3’** | **Annealing temperature** |
| --- | --- | --- |
| APC_F | CAAGGAAGTATTGAAGATGAAGCTATG | 62°C |
| APC_R | CCATAAGAACGGAGGGACATT | 62°C |
| BCL2_F | GAAGTCTGGGAATCGATCTGG | 62°C |
| BCL2_R | TCCCATCAATCTTCAGCACTC | 62°C |
| CCND1_F | CCCGCACGATTTCATTGAAC | 62°C |
| CCND1_R | GGCGGATTGGAAATGAACTTC | 62°C |
| CDKN1C_F | CTGACCAGCTGCACTCG | 62°C |
| CDKN1C_R | CTCAGGCGCTGATCTCTTG | 62°C |
| CTNNB1_F | TGGTTAAGCTCTTACACCCAC | 62°C |
| CTNNB1_R | CACGAACAAGCAACTGAACTAG | 62°C |
| EZH2_F | CACTCCTTTCATACGCTTTTCTG | 62°C |
| EZH2_R | ATGCTGGTAACACTGTGGTC | 62°C |
| GAPDH_SYBR_F | GAAGGTGAAGGTCGGAGTC | 60°C |
| GAPDH_SYBR_R | GAAGATGGTGATGGGATTTC | 60°C |
| GSK3B_F | GGTCTATCTTAATCTGGTGCTGG | 62°C |
| GSK3B_R | TGGATATAGGCTAAACTTCGGAAC | 62°C |
| MCL1_V1_F | GGCAGTCGCTGGAGATTATC | 60°C |
| MCL1_V1_R | TTCCGAAGCATGCCTTGGAA | 60°C |
| MCL1_V123_F | CGCCCTAAAACCGTGATAAAG | 62°C |
| MCL1_V123_R | CCGATTACCGCGTTTCTTTTG | 62°C |
| MYC_F | CAGATCAGCAACAACCGAAA | 60°C |
| MYC_R | GGCCTTTTCATTGTTTTCCA | 60°C |
| PTEN_F | AGTCCAGAGCCATTTCCATC | 62°C |
| PTEN_R | CCTTTTGTTTCTGCTAACGATCTC | 62°C |
| TP53_F | CCTCAGCATCTTATCCGAGTG | 62°C |
| TP53_R | ACATGTAGTTGTAGTGGATGGTG | 62°C |
| IL6ST_F | GCAACATTCTTACATTCGGACAG | 60°C |
| IL6ST_R | TCCCACTCACACCTCATTTTC | 60°C |
| STAT3A_F | AGGTCAACTCCATGTCAAAGG | 62°C |
| STAT3A_R | AGGTCAACTCCATGTCAAAGG | 62°C |
| STAT3B_F | CATCCTGAAGCTGACCCAG | 62°C |
| STAT3B_R | CATCAATGAATCTAAAGTGCGGG | 62°C |
